# Psychedelics: Alternative and Potential Therapeutic Options for Treating Mood and Anxiety Disorders

**DOI:** 10.3390/molecules27082520

**Published:** 2022-04-14

**Authors:** Henry Lowe, Ngeh Toyang, Blair Steele, Justin Grant, Amza Ali, Lorenzo Gordon, Wilfred Ngwa

**Affiliations:** 1 Biotech Research and Development Institute, University of the West Indies, Mona 99999, Jamaica; lowebiotech@gmail.com (H.L.); ngeh.toyang@flavocure.com (N.T.); justin@psyence.com (J.G.); amza@psyence.com (A.A.); 2 Vilotos Pharmaceuticals Inc., Baltimore, MD 21202, USA; 3 Flavocure Biotech Inc., Baltimore, MD 21202, USA; 4 Institute of Human Virology (IHV), University of Maryland School of Medicine, Baltimore, MD 21202, USA; 5 The Psyence Group, Toronto, ON M5J 2J1, Canada; 6 Caribbean School of Medical Sciences, Kingston 99999, Jamaica; lorenzogordon2011@yahoo.com; 7 Brigham and Women’s Hospital, Dana-Farber Cancer Institute, Harvard Medical School, Boston, MA 02215, USA; wngwa@bwh.harvard.edu; 8 Johns Hopkins University School of Medicine, Baltimore, MD 21218, USA

**Keywords:** psilocybin, psychedelic, neuropharmaceuticals, neurotherapeutics, addiction, anxiety, depression, cancer, psychopharmacology

## Abstract

The word “psychedelic” (psyche (i.e., the mind or soul) and delos (i.e., to show)) has Greek origin and was first coined by psychiatrist Humphry Osmond in 1956, who had been conducting research on lysergic acid diethylamide (LSD) at the time. Psychedelic drugs such as *N*,*N*-DMT/DMT (*N*,*N*-dimethyltryptamine), 5-MeO-DMT (5-methoxy-*N*,*N*-dimethyltryptamine), LSD (lysergic acid diethylamide), MDMA (3,4-methylenedioxymethamphetamine) and psilocybin have had significant value as an entheogen in spiritual, religious (shamanic) and sociocultural rituals in Central and South American cultures for thousands of years. In the 1960s, the globalization of these drugs and their subsequent spread outside of their indigenous, old-world cultures, led to the subsequent implementation of strict drug control laws in many Western countries. Even today, psychedelics are still classified as Schedule I drugs, resulting in a still lingering negative stigmatization/perception, vilification, and ultimate criminalization of psychedelics. This controversy still lingers and still limits scientific research and full medical acceptance. For many years up until recently, the spiritual, religious and medicinal value of these drugs could not be explored in a scientific context. More recently, a second wave of psychedelic research is now focusing on psychedelics as neuropharmaceuticals to treat alcohol and tobacco addiction, general mood and anxiety disorders and cancer-related depression. There is now a vast array of promising evidence-based data to confirm the years of anecdotal evidence of the medicinal values of psychedelics. Natural therapeutic alternatives such as psychedelic drugs may provide a safe and efficacious alternate to conventional drugs used to treat mood and anxiety disorders. In a Western context in particular, psychedelic drugs as therapeutic agents for mood and anxiety disorders are becoming increasingly of interest amidst increasing rates of such disorders globally, changing social constructions, the implementation of government regulations and increasing investment opportunities, that ultimately allow for the scientific study to generate evidenced-based data. Alternative psychotherapeutic interventions are gaining interest also, because of their low physiological toxicity, relatively low abuse potential, safe psychological effects, and no associated persisting adverse physiological or psychological effects during and after use. On the other hand, conventional psychotic drugs and anti-depressants are becoming less favorable because of their adverse side effects. Psychedelic neuropharmaceutical interventions may with medical oversight be the solution to conventional psychiatric disorders such as depression and anxiety, and an alternative to conventional psychiatric treatment options. This paper will review the therapeutic potential of psychedelic drugs as alternative therapeutic options for mood and anxiety disorders in a controlled, clinical setting, where the chances of adverse psychological episodes occurring are mitigated.

## 1. Introduction

### 1.1. Change in Status Quo

Up until recently, psychedelics were only perceived as part of the illicit recreational drug culture despite their wide use in psychiatry before 1967 [1]. Subsequent globalization of this culture led to the reclassification of psychedelic drugs into the Schedule I class of the United Nations Convention on Drugs in 1967 [1], and the prohibition of psychedelic drugs. Though not as prominent as before, this negative stigmatization still lingers, but is dwindling due to a change in status quo, propelled by scientific evidenced-based data confirming the therapeutic potential of these drugs against multiple disorders. Further long-term rigorous studies are required before psychedelics can become a mainstay in research and clinical settings. 

Generally, there is a shift toward natural medicinal alternatives. Amidst increasing rates of depression worldwide now compounded by the COVID-19 pandemic, there is a need for alternative options to conventional mood and anxiety disorders as patients are being prescribed more anti-anxiety, anti-insomnia, antidepressant prescription drugs to cope with the pandemic [2]. The most commonly prescribed antidepressants, selective serotonin reuptake inhibitors (SSRIs), the gold standard, produce severe side effects such as increased risk of suicide, sexual issues, increased risk for internal bleeding due to reduced capacity to clot, increased risk of drug interactions, potential risk to fetus, among others [3,4]. Other commonly prescribed anti-depressants such as tricyclic antidepressants produce severe side effects such as disturbances in heart rhythms, dizziness that can result in a fall, potential risk to fetus and increased risk of drug interactions [3,4].

The 2019 U.S. Food and Drug Administration (USFDA)-approval of SPRAVATO^®^, a ketamine analog developed by Johnson and Johnson for use in patients suffering from treatment-resistant depression, and the approval of “breakthrough therapy” statuses for psilocybin treatments for Major Depressive Disorder (MDD) by Compass Pathways Ltd. (in 2018) and the Usona Institute (in 2018), are also expected to spur the growth of psychedelic research.

Psychedelics, in particular, generally have low physiological toxicity, safe psychological responses, low addictive/dependence potential, low chance of neurological deficits after use and no associated persisting adverse physiological or psychological effects during or after use [5,6,7,8,9,10,11]. In addition to these properties, psychedelics produce only relatively minor side effects in comparison to commonly prescribed antidepressants [3,12], produce therapeutic effects in patients more quickly than commonly prescribed antidepressants that may take several weeks to produce effects [4] and may produces positive, long-lasting effects after only a single dose/therapy session [12]. This does not mean that psychedelic drugs are entirely risk-free. Psychedelics still have a high abuse potential, particularly in recreational settings. To replicate and confirm these findings, larger, longer-term studies with appropriately represented samples of a given population, and standardized dosages will be required. 

On this tangent, it should be noted that toxicities and potentials for abuse exist but vary among these psychedelic drugs. It is also important to note that patients with a family history of psychotic disorders and/or any degree of suicidality are less likely to benefit from psychedelic treatment and should be excluded from psychedelic therapy.

While not all psychedelic drugs may make their way into the medical mainstream (as in the case of LSD), they are still significant in the overall historical context of psychedelic use. Lead compounds identified in these psychedelic drugs may have the potential to be developed into pharmaceuticals for the treatment of mood and anxiety disorders. Now that potential lead compounds from psychedelic drugs have been identified, there are several following steps in the drug development process that involve validation of their potential, pre-clinical research, synthesis of the lead compound into an optimal form for delivery into the body, and ultimately clinical research. Other factors such as benefits, efficacies of these lead compounds, mechanisms of action, risks, adverse effects, drug interactions, toxicities, possible synergies between other compounds and cellular responses to other drugs including traditional, mainstay antidepressant drugs, should also be investigated.

US Food and Drug Administration (FDA)-approval of such psychedelic pharmaceuticals and substantiated clinical decision-making are strictly dependent upon the elucidation of the aforementioned factors and the generation of more evidence-based data.

### 1.2. Classification of Psychedelic Drugs

Psychedelic drugs typically under one of three classes. These include; (1) the tryptamines such as *N*,*N*-dimethyltryptamine (DMT) and its derivatives alpha-methyltryptamine (AMT), 5-methoxy-*N*,*N*-dimethyltryptamine (5-MeO-DMT) and 5-methoxy-*N*,*N*-diisopropyltryptamine (5-MeO-DIPT), psilocybin ([3-(2-dimethylaminoethyl)-1*H*-indol-4-yl] dihydrogen phosphate and its metabolite psilocin (4-hydroxy-*N*,*N*-dimethyltryptamine a.k.a 4-OH-DMT), (2) the Phenethylamines (PEA) such as MDMA, MDMA-like drugs such as *p*-methoxy methamphetamine (PMMA), mescaline and mescaline-derived compounds like TMA, DOM, DOET, DOI (2,5-dimethoxy-4-iodoamphetamine), and DOC (2,5-dimethoxy-4-chloroamphetamine), and (3) the lysergamides such as LSD, that have activity against both serotonin and dopamine receptors [13,14,15]. PEA psychedelics are characterized by a phenethylamine core structure with one or more hydrogen atoms in the structure replaced by another function group., whereas the lysergamides such as LSD are polycyclic amides of lysergic acid that are characterized by the presence of both phenethylamine and tryptamine groups with a carboxamide group attached to the eighth carbon atom of the structure. 

**Figure 1:**
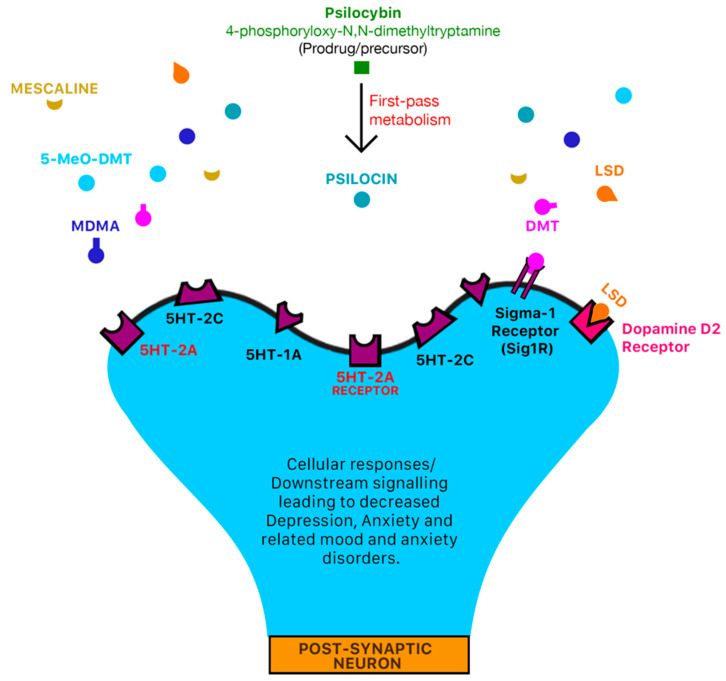
Mechanism of action of classic serotonergic drugs. 5-HT serotonin receptors are densely located in areas of the brain that are responsible for mediation of mood and anxiety disorders such as the pre-frontal cortex [18,24]. Classic serotonergic psychedelic drugs such as LSD, DMT, 5-MeO-DMT, mescaline, psilocybin and MDMA all have an affinity for serotonergic 5-HT receptors [17] which may mediate the psychotomimetic and pharmacological effects of psychedelic drugs. LSD may also interact with dopamine D2 receptors and trace-amine associated receptors (TAARs) to produce psychotomimetic and pharmacological effects [22]. DMT also interacts agonistically with the sigma-1 receptor (Sig1R) [22,23] and trace-amine associated receptors (TAARs) to produce anti-inflammatory and anti-oxidant effects [22]. In pre-clinical animal models, trace-amine associated receptors such as TAAR1 have been identified as a novel target for metabolic disorders, drug addiction, neurological and psychiatric diseases such as depression and schizophrenia [25,26,27,28].

**Figure 2:**
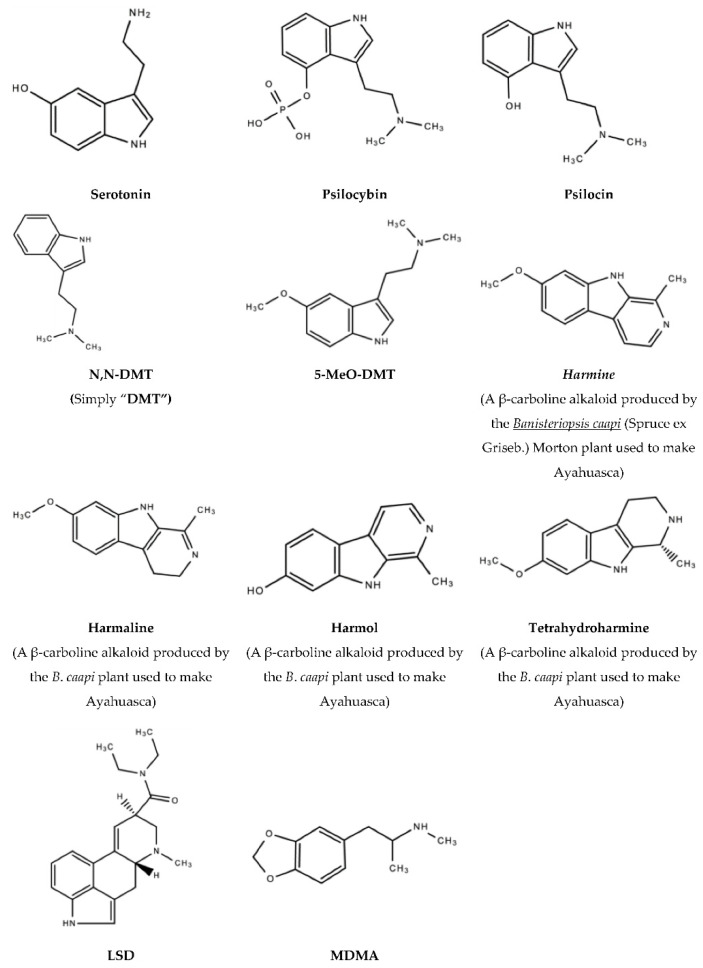
Serotonin, psychedelic drugs and derivatives of psychedelic drugs with therapeutic potential.

Tryptamine is an indolamine metabolite of tryptophan, an essential amino acid, and is characterized by an indole ring and a 2-aminoethyl group at the third carbon atom [16]. Tryptamine psychedelics such as DMT (and by extension, 5-MeO-DMT), and psilocybin (and by extension, Psilocin), very strongly resemble serotonin in chemical structure, as is the general case with serotonergic psychedelics. Classic psychedelic (serotonergic) drugs interact with the serotonin receptors (5-HT/5-hydroxytryptamine receptors) and their subtypes densely located within the brain [17,18]. Refer to Figure 1 below for the general mechanisms of action of serotonergic drugs and Figure 2 for the chemical structures of serotonin, psychedelic drugs and derivatives of psychedelic drugs with therapeutic potential. These receptors mediate emotions and moods such as anxiety and aggression, cognition, sex, learning memory, appetite along with other biological, neurological and neuropsychiatric processes [18,19]. 5-HT receptors are also located in the central and peripheral nervous systems [20,21]. Serotonin receptors are the target of multiple recreational and pharmaceutical drugs such as hallucinogens, empathogens, antipsychotics, antidepressants, antiemetics, antimigraine agents and anorectics [19]. It should also be noted that these drugs may also interact with other receptors to produce effects [22,23].

### 1.3. Treating Mood and Anxiety Disorders with Psychedelic Drugs

“Classical psychedelics” are so referred to because of their agonistic activity at the serotonin 2A receptor (5-HT_2A_) [29] and most importantly because of their sociocultural influence, prominence as recreational drugs, and frequent occurrence in scientific publications. Some classical psychedelics include ayahuasca, DMT, 5-MeO-DMT, LSD, MDMA (3,4-Methylenedioxymethamphetamine), Psilocybin and mescaline. It should be noted, however, that mescaline is not a serotonergic psychedelic but a phenethylamine psychedelic. The Serotonin Hypothesis proposed in the 1960s has played a significant role in the advancement of molecular/biological psychiatry. This hypothesis postulated that mood and anxiety disorders were the result of deficits in levels of serotonin in the brain [30], since it is generally accepted that 5-HT serotonin receptors are densely located in areas of the brain that are responsible for mediation of mood and anxiety disorders such as the pre-frontal cortex [18,24]. Based on this hypothesis, current anti-depressants are designed to increase serotonin levels and essentially reverse mood and anxiety disorders. It is generally accepted now in the scientific landscape, that multiple factors and combinations thereof, such as genetics, environment and other biological system (such as the norepinephrine (NE) and dopamine (DA) systems) –may also play a role in mental disorders [30].

Despite thousands of years of anecdotal evidence, psychedelic drugs are becoming increasingly popular, as their medicinal values in treating mental disorders are increasingly being elucidated. Psychedelic drugs such as ayahuasca, DMT, 5-MeO-DMT, LSD, MDMA (3,4-Methylenedioxymethamphetamine), and psilocybin have shown great promise in treating mood and anxiety disorders, neurodegenerative disorders, alcohol-use disorder, and various substance-use disorders, particularly in patients who are treatment-resistant and/or facing a terminal illness [6,7,12,31,32,33,34,35,36,37,38,39,40,41,42,43,44,45,46,47,48,49,50,51,52,53,54,55,56]. It is important that set (mindset) and setting (whether therapeutic or ritual), are prepared to facilitate/enhance a patient’s experience and therapeutic outcome [57,58].

More recently in the 20th century, the globalization of psychedelics beyond their native cultures and countries, led to the formation of strict national drug control laws in many Western countries [59]. This led to a growing negative social construction of psychedelics and the implementation of strict government regulations that hindered the scientific study of these drugs. Due to changes in social construction, and amendments to government policy, there has been increasing opportunities for investment into the psychedelic industry and for scientific research. 

The significant barrier that negative stigmatization poses to implementing psychedelic treatments represents an important hurdle for academics to be aware of. Stigma is the negative social attitude attached to a characteristic of an individual, in this instance as a user of a substance such as a psychedelic agent, and how it is perceived by the rest of the population. This is particularly important because in most parts of the world these agents are still not available for legal usage. It therefore represents a hurdle to both the prescriber and the consumer of these substances but the availability of evidence of good quality will ultimately help to address this obstacle. 

Though the molecular mechanisms of action of these psychotherapeutic interventions are still being elucidated, evidence suggests that neuronal changes in brain structure and function also accompany the therapeutic effects of these drugs such as increased personal meaningfulness, increased mindfulness, increased introspection and a positive change in one’s outlook on life [60].

### 1.4. Psilocybin

Psilocybin mushrooms have played a significant role as an entheogen in mushroom-worshipping ceremonies in old-world Aztec and Mayan cultures some 6000 to 7000 years ago, so much so that the Aztecs even referred to magic mushrooms as *teonanactl* (“God’s flesh”) [45,61,62]. Although cave drawings depict the use of mushrooms in religious ceremonies, the earliest written recorded evidence of the use of psilocybin-producing mushrooms is in the *Florentine Codex*, a manuscript of ethnographical research of Mexican and Mesoamerican cultures, compiled between 1529 and 1579 by a Spanish Franciscan friar, Bernardino de Shagún [63]. Harvard botanist, Richard Evans Schultes also documented the ritual use of the aforementioned mushrooms in 16th century Mesoamerican cultures and identified the psilocybin-producing mushroom species used as *Psilocybe caerulescens*, *Panacolus campanulatus*, and *Strophia cubensis* [63]. 

Psilocybin is now the most studied psychedelic drug and many studies now confirm psilocybin-assisted therapy as a promising adjunct to psychotherapy for the treatment of pain and inflammation, cluster headaches, and mood and anxiety disorders such as Major Depressive Disorder, Post-Traumatic Stress Disorder (PTSD), Generalized Anxiety Disorder (GAD), Obsessive-Compulsive Disorder (OCD), Severe Existential Depression [6,31,45]. 

In 2018 Compass Pathways Ltd. (London, UK) received FDA approval of “breakthrough therapy” status for a psilocybin treatment they developed for treatment-resistant depression [30]. In the following year, the Usona Institute received also FDA “breakthrough therapy” status for a psilocybin treatment for major depressive disorder (MDD) [31]. 

### 1.5. *N*,*N*-DMT/DMT (The “God/Spirit Molecule”)

DMT is a naturally occurring indole alkaloid found in major plants such as *Acacia (Acacia catechu* (L. f.) Willd., *Acacia chundra* (Rottler) Willd, *Acacia mellifera* (M. Vahl) Benth), *Virola*, *Psychotria*, *Phalaris*, *Delosperma* and *Desmodium* and produced endogenously (extra-cerebrally) in animals in trace amounts as metabolic byproducts [64,65,66,67].

Despite the fact that the psychedelic/physiological effects of DMT and the symptoms of schizophrenia differ, several inconclusive studies conducted in the 1960s—1970s suggest that endogenous DMT, a possible “schizotoxin” [68], may play a role in psychosis and schizophrenia [69]. This was evidenced by increased urinary excretion of DMT (without hallucinations) in patients with slowly or rapidly cycling manic-depressive illness who were experiencing a psychotic episode [66,70]. In contrast to injections of DMT that produce immediate psychoactive effects, endogenous DMT does not seem to produce hallucinations, at least not in patients with slowly or rapidly cycling manic-depressive illness [66]. While studies suggests that DMT levels produced by normal, healthy controls and schizophrenic patients did not differ significantly [68,71], another study reported less urinary excretion of DMT in psychotic depressives when compared to neurotic and normal controls [70]. As a possible “schizotoxin”, DMT may have been of particular importance because some studies suggested a positive correlation between elevated DMT levels in psychiatric patients, mood and anxiety disorders (such as stress) and positive symptoms of psychosis and schizophrenia [72]. Normal, healthy patients also experienced positive symptoms of psychosis after being treated with exogenous DMT [72]. On these bases, it has been suggested that endogenous DMT may even mediate the possible symptoms observed in schizophrenic/psychotic patients [72]. The transmethylation theory of schizophrenia postulates that schizophrenia is caused by the accumulation of methylated indolealkylamines such as DMT, 5-MeO-DMT, and bufotenin (5-hydroxy-*N*,*N*-dimethyltryptamine) due to decreased MAO activity [71]. This schizophrenic activity resembles the experimental psychosis produced after drinking ayahuasca, rich in DMT [71]. There is, however, very little scientific evidence to support the transmethylation hypothesis and it was ultimately discarded [68,73]. However, this hypothesis had set the pace for molecular psychiatry. Instead, the serotonin hypothesis is now the foundation of the neurobiological and neurochemical studies of mood and anxiety disorders, despite the lack of full elucidation of how the serotonergic system functions in the pathophysiology these disorders [74].

DMT is a part of a class of serotonergic drugs that mediate psychological effects via 5-HT_1A_- and 5-HT_2A_ agonism [69,75,76,77], to produce short, episodic hallucinations [76,78]. It also has very high affinity for the 5-HT_2C_ receptor [79]. When ingested orally, DMT is degraded by visceral monoamine oxidase (MAO); this renders it inactive [80]. As a result, it must be taken with a monoamine oxidase inhibitor (MAOI), that prevents the degradation by MAO and renders it psychoactive [80,81]. MAOIs may also enhance the interaction of DMT with 5-HT_1A_ and 5-HT_2A_ receptors increasing the intensity and duration of psychoactive effects [79,81]. MAOIs such as isocarboxazid (Marplan^®^), phenelzine (Nardil^®^), selegiline (Emsam^®^) and tranylcypromine (Parnate^®^), were the very first anti-depressant pharmaceutical drugs developed to prevent depression by preventing the degradation of the neurotransmitters norepinephrine, serotonin and dopamine by monoamine oxidase [82].

It has been hypothesized that DMT’s psychotherapeutic potential, particularly its antistress, antioxidant, anti-amnesic and anti-inflammatory properties, may, at least in part, be the result of sigma-1 receptor (Sig-1R) agonism [83,84,85]. Further studies are required to confirm this hypothesis. Essentially, the sigma-1 receptor protects the cells of the body against hypoxia and oxidative/endoplasmic reticulum (ER) stress via activation of an antioxidant response [83,86]. Activation of the sigma 1 receptor has also been linked to enhancement of neuroimmunomodulation, neuroplasticity and neuroprotection, in addition to promotion of cell survival [85]. The anti-amnesic properties of DMT allows PTSD patients to retrieve traumatic memories. One possible anti-PTSD mechanism of action is to allow said patients to face traumatic memories, combat them and overcome them [85]. Enhancement of neuroimmunomodulation via activation of sigma 1 receptor suggests that DMT may also be used to treat diseases characterized by cellular memory dysregulation such as cancer, diabetes, autoimmune and neurodegenerative diseases [85]. DMT also plays a role in nervous system signaling [87]. 

It has also been suggested that endogenous DMT produced anxiolytic effects, and a calm and relaxed mental state, rather than psychotic symptoms, through an interaction with trace amine receptors [69]. Contrary to other psychedelics, it has been reported that DMT is the only classic hallucinogen that does not have the capability to induce tolerance to the psychological effects that it produces [75].

DMT is reported to have limited neurotoxicity and adverse effects [87]. It is important to note that the prevention of the breakdown of DMT in the body due to MAOIs may result in serious interactions of DMT with drugs such as antidepressants, psychiatrics medications, weight loss medications, St. John’s Worts, and many other medications [88]. 

### 1.6. Ayahuasca

The word “ayahuasca”, where “aya” translates to “soul” or “ancestor” and “huasca”/”wasca” translates to “rope” or “vine”, has roots in the Quechua language native to cultures of the Peruvian Andes [57]. The word essentially translates to “vine of the soul”, a testament to its hallucinogen properties [57].

Ayahuasca is a popular entheogenic brew made from psychoactive South American plants and used in spiritual, religious and sociocultural rituals as far back as pre-Colombian times, as evidenced by a chemical analysis of shamanic paraphernalia dating back an estimated 1000 years ago [89,90]. The decoction is made from the vine of the *B. caapi*, the shrub of the *Psychotria viridis* Ruiz and Pav. Plant—also known as chacruna—(or a substitute) and in some cases, other plants [91]. In addition to shamanistic and divination uses in central and south America, ayahuasca was also used to diagnose and treat multiple disorders, used as an aphrodisiac, and also used in hunting and warfare [92].

The psychoactive properties of the brew are attributed to DMT (from the *P. viridis*, *Psychotria carthagenensis* Jacq. or *Diplopterys cabrerana* *(Cuatrec.) B. Gates plant* [88,92] and *B. caapi*-produced MAOIs [88,93], such as the beta-carboline harmine [80]. In addition to harmine, other major alkaloids produced by *B. caapi* include tetrahydroharmine and harmaline [80,94]. To reiterate, when DMT is ingested orally, it is degraded by visceral MAO and this renders it inactive [80]. *B.* *caapi*-produced MAOIs prevent this degradation and the brew retains psychoactive properties [80,81]. In other words, the beta-carbolines increase the bioavailability of DMT [80,95]. The anti-depressant effects of ayahuasca are attributed primarily to DMT and to the *B*. *caapi* β-carboline alkaloids present [94]. A chemical analysis of shamanic paraphernalia used in pre-Colombian, South American shamanic rituals some 1000 years ago, point to the use of DMT and harmine, possibly used to make ayahuasca [90,96]. DMT was first synthesized in 1931 by Richard Helmuth Frederick Manske, a German-Canadian chemist [97,98]. Brazilian chemist and microbiologist Oswaldo Gonçalves de Lima is often credited for being the first to extract DMT (then referred to as “Nigerine”) in 1946 from the root of the *Mimosa hostilis* (C. Mart.) Benth. plant [98]. However, the first groundbreaking DMT studies were conducted by Dr. Rick Strassman in 1994 who studied the dose-response effects of intravenously-administered DMT in experienced hallucinogen users [60,78]. In addition, Dr. Strassman reported DMT’s ability to cross the blood–brain-barrier, suggesting that it may be an essential component to normal brain physiology [60,78].

Anti-depressant and anxiolytic properties of DMT have been described in an open-label study on psychiatric inpatients with a current depressive episode, which evaluated the effects of a single dose of ayahuasca [33,35]. Outcome measures from this study reflected statistically significant reductions in depressive scores after administration of ayahuasca [36]. Other controlled trials have also confirmed the potential of ayahuasca in treating treatment-resistant depression [32,33,99], and major depressive disorder (MDD) [32,33], anxiety [69], panic-like episodes [100], and hopelessness [100]. Ayahuasca may also have potential to treat suicidality [101] as confirmed by a randomized placebo-controlled trial on patients with treatment-resistant depression.

The positive effects of ayahuasca on the psychometric measures of anxiety, panic-like and hopelessness [99], increased satisfaction with life and ego dissolution [102] have also been explored. Ayahuasca has also demonstrated its ability to induce mindfulness-based stress reduction (MBSR) [102,103]. One study has even reported the potential of ayahuasca in the treatment and remission of treatment-resistant depression via modulation of cortisol to control stress levels [104].

The anti-oxidant and anti-inflammatory properties of ayahuasca may be attributed to the presence of DMT and harmine from the plant preparations [57,105]. Harmine has also demonstrated neuroprotectivity such as the reduction in levels of inflammation, cellular oxidative stress and excitotoxicity, cognitive-enhancing effects, improved memory and learning capabilities, and overall better neuropsychological function [105]. Furthermore, harmine is also shown to increase levels of brain-derived neurotrophic factor (BDNF), a protein responsible for hippocampal neurogenesis, survival of neurons, and neuroplasticity [57,94,105]. This suggests that the sustained anti-depressant effects of ayahuasca may be due to modulation of brain plasticity [94]. Brain regions such as the left nucleus accumbens, right insula and left subgenual area, areas involved in the regulation of mood and emotions, were also shown to experience increased blood perfusion after ayahuasca administration [99].

In addition to treating mood and anxiety disorders, ayahuasca may also have potential to treat substance dependence and to prevent relapse [85,106,107]. A preliminary observational study investigating the use of ayahuasca-assisted treatment for substance use disorder and stress in a rural First Nations community in British Columbia, Canada, reported statistically significant reduction in alcohol, tobacco and cocaine use [107]. However, these results were not consistent with cannabis and opiate use [107]. 

Similar to other psychedelics, another reported mechanism of action of ayahuasca is via modulation of the functional connectivity and activity of the default mode network (DMN), particularly deactivation of some parts of the DMN [108]. The DMN is more metabolically active during rest (mediation and sleep) than when carrying out goal-directed tasks [108,109,110]. Changes in functional connectivity within the DMN are associated with an altered state of consciousness (including mediation, mind-wandering and sleep) [111,112,113,114,115,116,117]. A study by Brewer and colleagues reported that during different types of meditations such as concentration, loving-kindness, and choiceless awareness, the DMN was relatively deactivated [118]. The DMN is also associated with the retrieval of autobiographical memory, mediation of social interactions, planning for the future, facilitating vicarious experiences through others, states of unconstrained cognition, self-referential processing, and internal and external awareness during wakefulness [110,115,119].

### 1.7. Ayahuasca Tourism

Ayahuasca tourism is a growing, multimillion dollar industry in Central and South America, particularly in the Peruvian Amazon [120,121,122,123,124]. Since the 1970s psychedelic era, tourists having been flocking retreat centers for spiritual healing, personal development and for the healing of mental illnesses and traumas [120,125]. As psychedelics are becoming more globalized and government restrictions loosen, we will continue to see the commercialization and exploitation of old-world psychedelic traditions, despite the fact that, as a result, many of these indigenous cultures and traditions will lose their authenticity, somewhat [120,126]. 

Although Peru may be considered as the ayahuasca capital of the world, retreats are emerging in other countries such as Costa Rica—with an estimated 20 official ayahuasca facilities, Brazil, Colombia, El Salvador, Ecuador, Bolivia, Mexico, North America, and several European countries such as Holland and Spain [125,127,128,129,130,131,132]. Still yet, many countries have not yet legalized psychedelics.

### 1.8. 5-MeO-DMT (Popularly Referred to as “Toad Venom”)

5-MeO-DMT is a naturally occurring indoleamine hallucinogen of the tryptamine class, classified as a non-selective serotonin (5-HT-1-A/5-HT-2A/C) receptor agonist [133,134,135]. 5-MeO-DMT is a less popular cousin of DMT (*N*,*N*-dimethyltryptamine), that is structurally similar, but has additional atoms attached to its structure. Along with 5-methoxy-*N*,*N*-diisopropyltryptamine (5-MeO-DiPT), 5-methoxy-*N*,*N*-diallyltryptamine (5-MeO-DALT) and DMT, 5-MeO-DMT is one of the most prevalent tryptamines [136]. 

5-MeO-DMT was first synthesized in 1936, and isolated from the plant *Dictyoloma incanescens* DC. and *Anadenanthera peregrina* (L.) Speg. seed in 1959 [137,138]. However, it is most notably associated with the Sonoran Desert Toad (also known as the Colorado River Road (*Bufo alvarius/Incilius alvarius*), native to northwestern Mexico and southwestern states of America [54,139,140]. The parotid glands of the *B. alvarius* toad secrete a milky-white toxin that contains the psychedelic substance [141]. The toxin of this toad contains 5-MeO-DMT and a closely related active metabolite, bufotenine, both of which have significant value as an entheogen in spiritual, religious (shamanic) and sociocultural rituals in Central and South American cultures [135,139,142]. Polymorphic cytochrome P450 2D6 (CYP2D6) is the enzyme responsible for the conversion of 5-MeO-DMT to bufotenine via o-demethylation [135]. It is also rendered orally inactive via deamination by visceral monoamine oxidase (MAO) when ingested orally [135]. MAOIs such as harmaline enhance the psychoactive effects of 5-MeO-DMT, possibly via enhancing the interaction of 5-MeO-DMT with 5-HT_1A_ and 5-HT_2A_ receptors [81,143,144].

Yopo snuff, a popular botanical 5-MeO-DMT preparation made from the seeds of the *A. peregrina* (L.) Speg. (yopo, jopo, parica, cohoba tree) tree, may have been an earlier development to the use of 5-MeO-DMT from Sonoran Desert Toads [145]. Although the drug is most often sourced from *A. peregrina* (yopo or cohoba) and *Virola theiodora*, other plant species such as *P. viridis* and *Mimosa tenuiflora* Benth. syn. *M. hostilis,* (C. Mart.) Benth. also known as jurema preta, calumbi (Brazil), tepezcohuite (México), carbonal, cabrera, jurema, black jurema, and binho de jurema also produce 5-MeO-DMT [140].

Recently unearthed evidence such as paraphernalia (ceramics and snuff tubes) suggest that pre-Columbian cultures (~3000–4000 years ago) in the Caribbean and South America utilized seeds of the native tree *A. peregrina* (L.) Speg. as an entheogen in religious ceremonies [146,147]. The first, official written accounts of the hallucinogenic properties of *A. peregrina* bean-snuff was reported by Friar Ramon Pane who was commissioned by Christopher Columbus to explore Hispaniola [148].

In addition to the entheogenic properties of 5-MeO-DMT, its therapeutic potential has also been recognized for the treatment of mood and anxiety disorders such as depression, anxiety, PTSD and drug addiction [50,51,52,53]. GH Research is currently conducting a clinical trial investigating the safety and psychoactive effects of GH001 (that contains 5-MeO-DMT) in healthy volunteers [149].

A study by Uthaug and colleagues investigated the effects of 5-MeO-DMT against mood and anxiety disorders. In a follow-up 4-weeks after administration of a single inhalation of the drug in the form of vapor from dried toad secretion, Uthaug and colleagues found increased ratings of satisfaction with life and convergent thinking, increased ratings of mindfulness, and decreased ratings of depression, anxiety and stress [141]. In another study by Uthaug and colleagues, it is reported that inhalation of vaporized synthetic 5-MeO-DMT also produced significant reductions in stress biomarkers (such as cortisol) and ratings of stress and anxiety [141]. 5-MeO-DMT may also have anti-inflammatory properties [141]. 

In another study, 5-MeO-DMT also demonstrated anti-depressant and anti-anxiolytic properties with low addictive potential and no adverse physical or psychological effects [51,140]. Participants of this study also reported mystical-type experiences, higher ratings of spiritual significance and higher ratings of the meaningfulness of the 5-MeO-DMT experience [51,140]. 

### 1.9. Lysergic Acid Diethylamide (LAD/LSD)

Up until the turn of the century, LSD was the most studied psychedelic drug [6]. LAD/LSD is an abbreviation for the German word “Lysergsäurediethylamid” [150,151]. LSD is an atypical hallucinogenic drug because it interacts with both serotonergic and dopaminergic receptors to produce psychoactive effects [13,14,15]. Other compounds in this class (including derivates of LSD) also have agonist and/or antagonistic activity for serotonin and dopamine receptors [152,153,154,155,156]. 

LSD was first synthesized in 1938 by Albert Hoffman of Sandoz Laboratories (Basel, Switzerland) while trying to synthesize a respiratory and circulatory stimulant from ergotamine, derived from the *Claviceps purpurea*, an ergot fungus [151,157]. This is likely the first discovery of an ergot alkaloid derivative with medical value. In 1947, Sandoz began marketing and distributing LSD as a psychiatric drug for the treatment of neurosis and alcoholism [37,38,39,40,41,158,159,160,161]

Other studies investigated the therapeutic potential of LSD (and derivatives, thereof) to treat diseases characterized by chronic inflammation such as Alzheimer’s disease, schizophrenia, multiple sclerosis, atherosclerosis and rheumatoid arthritis, criminal behavior, sexual perversions, autism, verbal behavior, frigidity, and other disorders such as cluster headaches, migraines, vascular headaches, phantom limb pain and addictions [42,43,44,162,163,164,165,166,167,168,169,170,171,172,173,174,175,176,177,178,179,180,181,182].

### 1.10. 3,4-Methylenedioxymethamphetamine (MDMA/“Ecstasy”)

MDMA was legally prescribed as a drug in the US (1970s to 1985) and Switzerland (1988–1993) [183]. Before the criminalization of MDMA in 1985 following a rise in its recreational use, psychiatrists utilized MDMA as a therapeutic adjunct to psychotherapy [184]. More recently, a 2011 study by Mithoefer and colleagues completed the first clinical trial evaluation of MDMA for the treatment of PTSD. They investigated the safety and efficacy of ±3,4-methylenedioxymethamphetamine-assisted psychotherapy in subjects with chronic, treatment-resistant posttraumatic stress disorder (PTSD) [184]. Researchers also reported no clinically significant drug-related adverse events, neurocognitive effects, or increases in blood-pressure [47,184]. In comparison to a placebo group, scores from the clinician-administered PTSD scale, the primary outcome measure, were significantly reduced from baseline [180]. Other studies report similar results [49,55,183,185].

A first-of-its-kind study assessing the safety and tolerability of MDMA-assisted psychotherapy in patients with alcohol use disorder also showed promise, despite only being in preliminary stages [47,48]. 

## 2. The Economic Value of Psychedelics

The psychedelics market is now a burgeoning market. This is primarily owed to increasing prevalence of mental health disorders, novel therapeutic opportunities, novel technologies and growing evidenced-based data that now support the thousands of years of anecdotal evidence in traditional medicine, and more recently, multiple pre-clinical and clinical studies. 

According to a report by Data Bridge Market Research across of forecast period of 2020–2027, the U.S. psychedelics drugs market is expected to grow to USD 6859.95 million by 2027 from USD 2077.90 million in 2019, at a compound annual growth rate (CAGR) of 16.3% [186]. According to another report by Data Bridge Market Research across a forecast period of 2020–2027, the European psychedelics drugs market is expected to grow to USD 361.13 million by 2027 at a CAGR of 15% [187].

## 3. Conclusions and Future Remarks

Mood and anxiety disorders are a great global burden. Amidst the issues facing traditional anti-depressants and anxiolytics such as unsafe toxicology, there is a need to explore novel treatment options. One such option is psychedelic-assisted psychotherapy. Psychedelic neuropharmaceuticals may provide therapeutic opportunities for patients suffering from mood and anxiety disorders, in addition to many other ailments. Said therapeutic interventions have demonstrated significant anti-depressive, anxiolytic, anti-addictive and anti-suicidal properties [35]. The increasing use of psychedelics globally, also directly correlates with an increase in “drug tourism”, as tourists are increasingly motivated and curious to try alternative options to treat health problems and other spiritual and personal issues [122,188]. South America, in particular, continues to attract tourists seeking the psychedelic experience [122]. Psychedelic retreats are also emerging in several countries including Jamaica, Canada, Netherlands, and certain states in the U.S. such as Washington, Colorado and California where some psychedelics are decriminalized. These countries and states are also at the forefront of cannabis tourism.

In order for psychedelic drugs to be fully integrated into modern healthcare, several limitations have to be addressed. Most importantly, there is still lingering controversy in science and government over the acceptability and use of psychedelic drugs. This extends from the era of psychedelic prohibition in the early 1970s. Secondly, neurobiological and physiological mechanisms of action still require clear elucidation. In the medical sciences, researchers are investigating active molecules in psychedelic drugs to target certain unmet medical issues. It is important to recognize that the issue is not whether one psychedelic is globally better than another. Instead, exactly analogous to the usage of diverse antidepressants for depression, we should regard this class of agents, also differing by chemical structure and mechanisms of action, as more suitable, because of varying clinical characteristics to a particular subset of patients than another. Treatment decisions will therefore eventually have to be tailored to the patient as we understand and define the place and purpose of each of these exciting agents in our therapeutic armamentarium. In addition, more rigorous evidence-based data is required before the full acceptance of psychedelics into modern medicine. The current literature on such studies is very limited in many aspects. As a result, more meticulous experimental designs are required in the future. Ideally, the lead compounds identified in these psychedelic drugs require further preclinical research, validation of their potential in a clinical setting, synthesis into optimal forms for delivery into the body, and ultimately an assessment of their efficacies in clinical research before they can make their way into modern medicine. US Food and Drug Administration (FDA)-approval of such psychedelic pharmaceuticals and substantiated clinical decision-making are strictly dependent upon the elucidation of the aforementioned factors and the generation of more evidence-based data. 

Lastly, education of all stakeholders to reduce the stigma associated with psychedelics as therapeutic agents will be extremely important for the appropriate clinical use of these medicines. 

## Data Availability

Data sharing is not applicable to this article. No new data were created or analyzed in this study.

## Sample Availability

Samples of the compounds are not available from the authors.
